# Inverted V Graft: Combination of Columellar Strut and Extension Graft

**DOI:** 10.3390/jcm15093291

**Published:** 2026-04-25

**Authors:** Meysem Yorgun, Erdinc Cekic, Ozgur Surmelioglu

**Affiliations:** 1 Otolaryngology Department, MMT American Hospital, Hatay 31500, Turkey; meysemyorgun@gmail.com; 2 Department of Otolaryngology, Haseki Education and Research Hospital, Health Science University, Istanbul 34096, Turkey; erdinccekic@gmail.com; 3 Department of Otorhinolaryngology, Faculty of Medicine, Çukurova University, Adana 01330, Turkey

**Keywords:** rhinoplasty, Rhinoplasty Outcome Evaluation, Inverted V graft, columellar strut

## Abstract

**Objective**: To explore the efficacy and outcomes of the ‘Inverted V graft’ technique, which synergistically combines a columellar strut and an extension graft. This innovative approach aims to provide enhanced structural support and improved aesthetic results in rhinoplasty procedures. **Methods**: Patients were observed postoperatively over a period of one year, with evaluations at designated intervals using the Rhinoplasty Outcome Evaluation (ROE) questionnaire to assess satisfaction. The closed preservation rhinoplasty method was employed under general anesthesia with all patients. The Inverted V graft was meticulously sutured in place, augmenting nasal structure and stability. **Results**: In a group of 19 participants, the study observed significant enhancements in patient satisfaction post-rhinoplasty, as indicated by ROE scores, with no age-related variation in outcomes. Surgical times averaged around 209.5 min, with an 18-month follow-up showing similar satisfaction improvements across both genders. **Conclusions**: The study demonstrates that the dual-purpose Inverted V graft technique significantly enhances structural support and aesthetic outcomes in rhinoplasty procedures. The use of this technique resulted in substantial improvements in patient satisfaction, as measured by the Rhinoplasty Outcome Evaluation (ROE) scores, indicating its effectiveness in achieving desired surgical results.

## 1. Introduction

The nose, a prominent feature on the face, plays a significant role in enhancing facial beauty [1]. The study of the human face and nose has long been a focal point for clinicians, scientists, and artists, exploring its role in communication, environmental interaction, personal identity through phenotypic features, and as an indicator of an individual’s health status, through both qualitative and quantitative analysis [2,3]. Furthermore, the dimensions of the nose serve as critical criteria for devising treatment strategies. The quest for the so-called perfect nasal form and the surgical interventions aimed at attaining it have been extensively documented in the literature [4].

The nasal form is distinctively indicative of one’s ethnicity, race, gender, and age. For surgeons engaged in nasal repair and reconstruction, understanding the nose’s specific shape, structural anatomy, and size is invaluable [5,6]. The variability of anthropometric measurements across different ages, genders, and ethnicities has been acknowledged, with numerous researchers documenting standard values that could act as benchmarks for various population groups [7,8]. The nasal architecture of the Turkish race shows distinct variations when compared with other ethnic groups. Particularly within the Turkish population, our research has highlighted a pronounced sexual dimorphism in nasal dimensions, revealing notable size discrepancies between males and females. It has been demonstrated that men possess larger vertical nasal measurements than women, while their horizontal nasal dimensions are narrower than those of females. This study underscores the unique nasal characteristics inherent to the Turkish ethnicity and provides critical insights for anatomical and surgical considerations based on gender differences [9].

In modern rhinoplasty surgery, tip projection and rotation are of paramount importance. The literature describes numerous cartilage grafts that alter tip rotation and projection. Among these, columellar strut and septal extension grafts are the most frequently preferred options [10]. While a free columellar strut graft provides advantages in positioning and rotation of the tip, a septal extension graft offers benefits in tip projection and stabilization of the tip to the septum. The technique we employ incorporates both extension and columellar strut characteristics, thereby ensuring a more secure rotation and projection. Additionally, we believe it reduces the risk of losing rotation and projection. In some patients where both extension and columellar strut are used together, contact between the two grafts can occur, manifesting as a bulge in the supratip area. Our technique facilitates easy adjustment of tip rotation and position, while also contributing to an increase in the projection of the nasal tip. It also ensures a more secure positioning of the tip to the septum.

In the present manuscript, we share our insights gained from employing this specific rhinoplasty technique within the Turkish context. Through the examination of a series of 19 cases, we offer protocols for crafting this particular graft, along with a comprehensive discussion on the technical nuances and possible challenges to enhance the efficacy of this approach.

## 2. Material and Methods

### 2.1. Ethical Approval and Informed Consent

This study was approved by the Training and Research Hospital Ethics Committee (reference number 78, dated 1 March 2024). Informed consent was obtained from all subjects and/or their legal guardians for the publication of identifying information/images in an online open-access publication. All methods were performed in accordance with the relevant guidelines and regulations.

### 2.2. Patient Selection

This study included patients undergoing primary rhinoplasty. All patients were discharged on postoperative day one and were scheduled for follow-up visits at one week post-surgery, where nasal packing and splints were removed.

### 2.3. Postoperative Care

Following the removal of tampons and splints, nasal taping was continued for one to two weeks. Patients received postoperative massage and care recommendations. Follow-up visits were scheduled at one month, three months, six months, and one year postoperatively. No complications were observed during the postoperative period. Satisfaction levels before and after the surgery were evaluated using the Rhinoplasty Outcome Evaluation (ROE) questionnaire. None of the patients required revision surgery.

### 2.4. Surgical Technique

All surgeries were performed under general anesthesia using the closed preservation rhinoplasty technique. After decontamination and sterile draping, local anesthetic was applied. A closed Dorsal preservation technique was chosen. Intercartilaginous and marginal incisions were made, followed by supraperichondrial and subperiosteal elevations. A wide elevation was preferred due to the use of a piezo device for osteotomies. Septal subperichondrial dissection was performed to correct deviations and remove the low septal strip. A cut was made to the perpendicular plate of the ethmoid bone. The septum was then stabilized at the anterior nasal spine (ANS). Osteotomies were completed using the previously described Mobile Bony Cap method with a piezo device. After completing the osteotomies, tip modification began with cephalic resection, identification of dome points, and placement of hemitransdomal sutures. Medial crural overlap was performed as needed.

**Figure 1:**
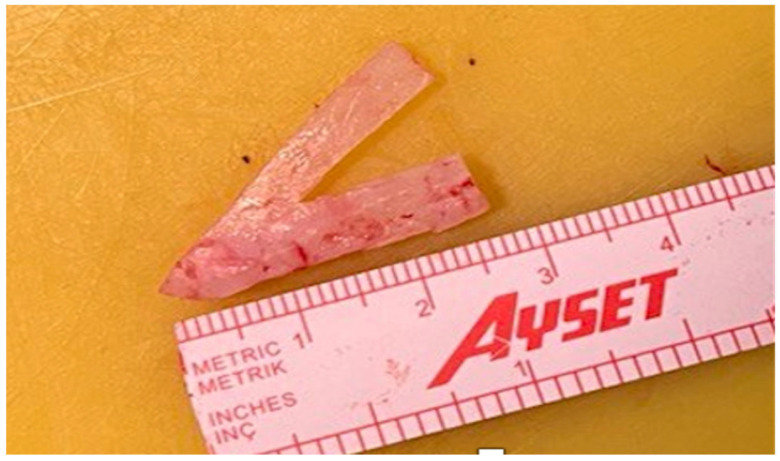
Inverted V graft.

The technique for fashioning the hybrid columellar strut and extension graft, referred to as the Inverted V graft, involves several precise steps to ensure optimal results. First, the need for a caudal strut graft is determined. This graft typically measures between 2.5 and 3 cm in length (Figure 1). The purpose of the caudal strut is to provide additional support to the nasal tip, preventing it from drooping postoperatively. Next, the need for a graft that extends from the dome to the caudal septum is assessed. This graft usually measures between 2 and 2.5 cm in length. This component of the graft provides support and stability to the nasal tip, ensuring proper projection and rotation. The graft is shaped into an Inverted V configuration. Sometimes, cartilage harvested from the septum base can be shaped this way. When harvesting cartilage from the septum base, the cartilage tends to be narrow at the caudal end and wider towards the cranial end. A piece is removed from the middle of the cranial part to form the legs of the Inverted V. Alternatively, two separate struts can be cut and sutured together in a V shape using PDS sutures. This ensures that the graft has the necessary strength and flexibility to support the nasal tip. The columellar portion of the graft is then positioned between the medial crura, acting as a columellar strut. This provides initial structural support to the nasal tip and prevents postoperative drooping. After positioning the columellar strut, the extension portion of the graft is sutured to the caudal septum. The desired level of extension and rotation is determined, and the graft is sutured in place accordingly. This ensures optimal nasal tip projection and rotation, allowing for better adjustment and stabilization compared to triangular or rectangular grafts. After the graft is securely placed, final adjustments are made to ensure proper alignment and fit. Any excess cartilage is trimmed to achieve the desired contour, and the graft is sutured in place with PDS sutures. The surgical site is then closed, and nasal taping is applied. Internal and external nasal splints are used to protect the graft and maintain its position during the healing process.

The domes were then joined, and excess was trimmed with a bistoury. Cap grafts or alar rim grafts were placed as required. Mucosal incisions were sutured with rapid vicryl 6/0. The procedure was concluded with nasal taping and the placement of internal and external nasal splints. The surgery duration was approximately 3–4 h. The Inverted V graft was first fixed like a columellar strut to the medial crura and then the back leg was attached to the septum at various angles. Therefore, it is recommended to first secure it to the medial crura before establishing the septal connection, although it can be fixed in the reverse order if desired. Mucosal incisions were closed with 6/0 rapid vicryl, and cartilages were sutured with PDS 5/0. Cap grafts were secured with 6/0 PDS, and depending on the strength of the cartilage connection to the septum, 4/0 PDS could also be used.

**Figure 2:**
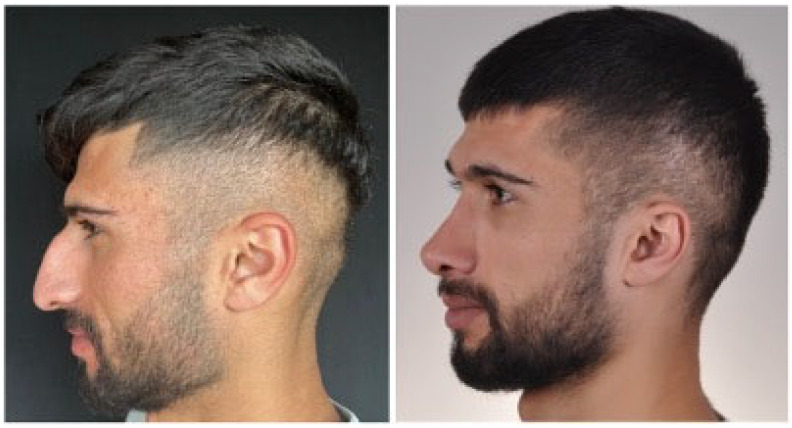
Pre- and postoperative Inverted V graft technique.

In order to ensure consistency and accurate comparison, standardized before and after photographs were taken for all patients (Figure 2). These photographs were captured at an equal distance, with the same lighting and background conditions. The views included frontal, lateral, and basal perspectives to provide a comprehensive visualization of the surgical outcomes. Additional patients and their results have been included to strengthen the findings and demonstrate the effectiveness of the Inverted V graft technique.

### 2.5. Statistical Analysis

Data were analyzed utilizing SPSS software (Version 27, SPSS Inc., Chicago, IL, USA). To determine the normality of distribution, we employed the Kolmogorov–Smirnov and Shapiro–Wilk tests, along with an assessment of histograms. Where variable distributions were normal, comparisons between the preoperative and postoperative ROE were made using the independent samples *t*-test. Conversely, the Mann–Whitney U test was applied for non-normally distributed data. A *p*-value of less than 0.05 was considered statistically significant.

## 3. Results

In the study we conducted a retrospective analysis of 19 patients presenting with developmental nasal deformities. Ethical approval for this retrospective study was obtained from the Training and Research Hospital Ethic Committee, documented under reference number 78, on 1 March 2024.

Within the study group comprising 19 individuals, the average age was calculated to be 27.0 ± 8.1 years, with a median age of 24 years, indicating that the majority of participants were young adults. The average Body Mass Index (BMI) of the participants was determined to be 21.9 ± 2.9. The average operation time was found to be 209.5 ± 26.4 minutes. The follow-up period averaged 18.0 ± 2.2 months. The follow-up period extended over an average of 18.0 ± 2.2 months, with the median follow-up demonstrating a slight skew towards the upper limit of 19 months, indicating a consistent short-term postoperative evaluation timeframe (Table 1).

In the study encompassing 19 patients, the Rhinoplasty Outcome Evaluation (ROE) scores revealed a substantial increase following the surgical procedure. The preoperative ROE scores were observed to be 3.8 ± 2.8 on average, with a median of 4.0 and an interquartile range of 2.0 to 6.0, reflecting a moderate baseline functional and aesthetic dissatisfaction among patients. Postoperatively, the ROE scores improved significantly to an average of 16.1 ± 2.3, median 16.0, with an interquartile range of 15.0 to 18.0, indicating a marked enhancement in patient satisfaction (Table 2).

The comparative analysis of Rhinoplasty Outcome Evaluation (ROE) scores before and after rhinoplasty across genders involved 7 female and 12 male patients. Preoperatively, females had an average ROE score of 3.86 ± 3.0 with a median score of 2.0, and males had a very similar average of 3.83 ± 2.7 with a median score of 4.0. Postoperatively, females achieved an average ROE score of 16.14 ± 1.2 with a median of 16.0, while males reported an average score of 16.08 ± 2.8 with a median of 16.0. Both groups displayed significant improvements in their ROE scores post-rhinoplasty with *p*-values of <0.001 for each (Table 3).

In the categorized age groups of the study, consisting of 10 participants aged 18–25, 6 in the 26–35 range and 3 within 36–45 years, the preoperative Rhinoplasty Outcome Evaluation (ROE) scores showed minimal variation across age groups. Specifically, the 18–25 age group had an average pre-op ROE score of 4.0 ± 3.0, the 26–35 age group also had a 4.0 ± 3.0 average, and the 36–45 age group had a slightly lower average of 3.0 ± 7.0; however, this difference was not statistically significant (*p* = 0.945). Postoperatively, ROE scores increased to 16.0 ± 3.0 for the 18–25 age group, 17.0 ± 2.0 for the 26–35 age group, and remained at 16.0 ± 2.0 for the 36–45 age group. These improvements were consistent and significant within each age category, as reflected by *p*-values of <0.001 across all groups. The data suggests that patient satisfaction, as measured by ROE, increased significantly after rhinoplasty regardless of age (Table 4).

In the BMI group below 18.5, there is one patient with an average delta ROE value determined to be 14. This group has exhibited the highest improvement in ROE post-surgery among all the groups studied. For the BMI group ranging from 18.5 to 24.9, there are 14 patients, with an average delta ROE value calculated to be 11.86 ± 3.18. This value is lower compared to the group with BMI below 18.5, indicating that individuals in this group experienced a moderate level of improvement in ROE post-surgery. In the BMI group of 25 and above, there are 4 patients, with an average delta ROE value found to be 13.25 ± 2.5. When compared to the group below 18.5, this group has shown slightly less improvement but a higher level of improvement than the group in the 18.5–24.9 range (Table 5).

**Figure 3:**
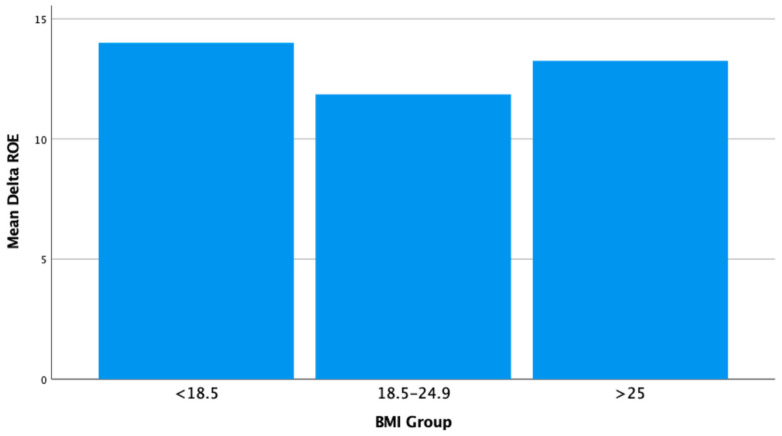
Comparison of ROE change according to BMI groups in rhinoplasty patients.

When examining the change in ROE by gender; it was calculated as 12.3 ± 3.2 for females (n = 12) and 12.3 ± 3.0 for males (n = 7). However, the difference in ROE change between genders was not found to be statistically significant (*p* = 0.967). Looking at the change in ROE according to BMI categories, in the <18.5 group (n = 1), the ROE change was calculated to be 14 ± 0. For the group in the 18.5−24.9 range (n = 14), the ROE change was observed to be 11.9 ± 3.2. In individuals with a BMI >25 (n = 4), the ROE change was determined to be 13.3 ± 2.5 (Figure 3). The difference in ROE change among BMI groups was not found to be statistically significant (*p* = 0.621).

## 4. Discussion

Rhinoplasty is a challenging procedure. The aim of the surgery is not merely to restore nasal function and a youthful appearance but also to enhance the quality of life. Over time, there has been a rapid shift from more invasive to less invasive procedures. Although the technical aspects of rhinoplasty are significant, patient satisfaction is the definitive factor in determining the success of the surgery [11]. Measuring patient satisfaction is a challenging task with no true standards established. JM Herruer et al. have examined the impact of psychological factors such as self-consciousness about appearance and expectations from the surgery [12]. It has been suggested that patients seeking surgery suffer distress due to their self-consciousness regarding their appearance [13]. Unexpected patient responses may occur even after successful surgical corrections because rhinoplasty can have a substantial psychological impact [14].

In 2000, Alsarraf et al. were the pioneers in developing a questionnaire deemed reliable for various plastic surgery procedures [15,16]. This questionnaire was later adapted by Arima et al. for individuals undergoing rhinoplasty and was termed the “Rhinoplasty Outcome Evaluation (ROE)” questionnaire [17]. The ROE scale comprises six questions that assess three qualitative dimensions: physical, psychological, and social. A postoperative score exceeding 80% is considered excellent, indicating a high level of patient satisfaction [18]. A recent study has posited that satisfying aesthetic expectations is more crucial than meeting functional expectations for patient satisfaction [19]. An improvement on the ROE scale by a minimum of 36 points is acknowledged as a significant gain [11].

The preference for the Inverted V graft over triangular or rectangular grafts is due to its superior ability to maintain both projection and rotational stability. Triangular or rectangular grafts may offer adequate projection but often lack the ability to secure the rotational position of the nasal tip as effectively. The Inverted V graft, by combining the features of both a columellar strut and a septal extension, ensures a more secure and stable nasal tip, reducing the risk of postoperative complications and the need for revision surgeries. The necessity of using an Inverted V graft instead of a SEG alone stems from its ability to provide comprehensive support to the nasal tip. The dual-function nature of the Inverted V graft addresses both the projection and rotational stability, which are critical for achieving long-lasting and satisfactory results in rhinoplasty. In our study, the utilization of this technique demonstrated significant improvements in patient satisfaction as measured by the ROE scores, underscoring the effectiveness of the Inverted V graft in delivering superior aesthetic and functional outcomes compared to traditional methods that rely solely on SEG. In some patients, the stitching of the columellar strut graft to the septal extension graft can result in a bulge between the septum and the columellar strut graft, which can be palpable in the supratip area [20].

This novel approach not only addresses the limitations inherent in existing rhinoplasty techniques but also offers a structural solution to enhance the long-term success of surgical outcomes. The Inverted V graft technique is advantageous for its less invasive nature leading to aesthetic improvement and the potential to reduce the need for revision rhinoplasty. Thus, it emerges as a valuable alternative for surgeons and patients in both aesthetic and functional rhinoplasty applications. When compared to techniques described in the literature, the Inverted V graft represents a significant innovation in preserving nasal tip projection and enhancing aesthetic results.

In our study employing the Inverted V graft technique for rhinoplasties, a significant increase was observed in the Rhinoplasty Outcome Evaluation (ROE) scores when comparing the preoperative and postoperative periods. This enhancement in ROE scores underscores the effectiveness of the Inverted V graft in addressing both aesthetic and functional patient concerns during rhinoplasty. Importantly, our findings indicated that the ROE scores remained consistent across varying patient demographics, such as gender and Body Mass Index (BMI), suggesting that the improvements in patient satisfaction attributable to the Inverted V graft technique are not influenced by these factors. This points to the broad applicability of the Inverted V graft technique in delivering improved patient outcomes irrespective of gender and BMI variations. While the combination of a septal extension graft (SEG) and a columellar strut (CS) is effective, we have observed occasional contour irregularities just above the nasal tip (infratip area) in some patients. This may be due to specific anatomical variations. Our Inverted V graft technique integrates both functions into a single structure, aiming to provide comprehensive support for nasal tip projection and rotation while minimizing such risks.

The focus of our research was the “Inverted V graft: Combination of Columellar Strut and Extension Graft” technique and its effects on the outcomes of rhinoplasty. The findings indicate that when compared to preoperative ROE scores, there was a fivefold increase, which is considered a significant indicator of the efficacy of our method. When compared with the study conducted by Stergiou et al., which observed an increase in ROE scores from 37.9 to 81.25 post preservation rhinoplasty in 58 patients, the improvement, although statistically significant, is lower than the results achieved with our surgical techniques [21]. This discrepancy highlights the potential of our technique to maximize both aesthetic and functional improvements.

In the study conducted by Khan et al., the open rhinoplasty approach was implemented in all patients, utilizing a septal cartilage graft in 69 patients, conchal cartilage graft in 12 patients, and rib cartilage in nine patients. The questionnaire was completed preoperatively and at a six-month follow-up period for all patients [22]. They recorded a mean preoperative ROE score of 30.5 and a mean postoperative ROE score of 79.5 at six months, which translates to an average increase of 133%. These figures are comparable to those reported by Alsarraf et al. [16], where the average preoperative score was 38.8 and the mean postoperative score was 83.3, representing an average increase of 115%. Similarly, Sena Esteves et al. [23] also reported comparable outcomes with a mean preoperative score of 32.78 and a mean postoperative score of 81.9, culminating in an average improvement of 150%.

The necessity of using an Inverted V graft instead of a SEG alone stems from its ability to provide comprehensive support to the nasal tip. The dual-function nature of the Inverted V graft addresses both the projection and rotational stability, which are critical for achieving long-lasting and satisfactory results in rhinoplasty. In our study, the utilization of this technique demonstrated significant improvements in patient satisfaction as measured by the ROE scores, underscoring the effectiveness of the Inverted V graft in delivering superior aesthetic and functional outcomes compared to traditional methods that rely solely on SEG.

There is some limitation of this study. The design of the study as a single-center trial may limit the generalizability of the findings to a wider population. Although the one-year follow-up period is sufficient for assessing immediate postoperative results, it may not reflect long-term satisfaction and the stability of the surgical outcomes. Additionally, the subjective nature of the Rhinoplasty Outcome Evaluation (ROE) questionnaire used to assess patient satisfaction could introduce bias; despite being a validated tool, it relies on self-reported data. The lack of a control group is another limitation, resulting in the absence of comparative outcomes with traditional rhinoplasty techniques.

## Figures and Tables

**Table 1:** Age and postoperative follow-up period in the study group.

	Study Group n = 19
**Age (years)**	27.0 ± 8.1
	24.0 [21.0; 33.0]
**BMI**	21.9 ± 2.9 21.0 [20.0; 24.0]
**Operation time**	209.5 ± 26.4 210.0 [185.0; 240.0]
**Follow-up (month)**	18.0 ± 2.2
	19.0 [18.0; 19.0]

Values are presented as mean  ±  SD and median [interquartile range]; n: number; BMI: Body Mass Index.

**Table 2:** Comparative analysis of ROE scores of patients before and after rhinoplasty.

	Study Group n = 19
**Pre-op ROE Score**	3.8 ± 2.8
	4.0 [2.0; 6.0]
**Post-op ROE Score**	16.1 ± 2.3
	16.0 [15.0; 18.0]
***p*-value**	**<0.001**

Values are presented as mean  ±  SD and median [interquartile range]; n: number; ROE: Rhinoplasty Outcome Evaluation. *p*-values were calculated with paired samples *t*-test.

**Table 3:** Gender-based comparison of preoperative and postoperative Rhinoplasty Outcome Evaluation scores.

	Female n = 12	Male n = 7
**Pre-op ROE Score**	**3.86 ± 3.0**	3.83 ± 2.7
	2.0 [2.0; 7.0]	4.0 [2.0; 6.0]
**Post-op ROE Score**	16.14 ± 1.2	16.08 ± 2.8
	16.0 [16.0; 17.0]	16.0 [15.0; 19.0]
***p*** **-value**	**<0.001**	**<0.001**

Values are presented as mean  ±  SD and median [interquartile range]; n: number. *p*-values were calculated with Mann–Whitney U test.

**Table 4:** Age group stratification of preoperative and postoperative Rhinoplasty Outcome Evaluation scores.

	18–25 Age (n = 10)	26–35 Age (n = 6)	36–45 Age (n = 3)	*p*-value
**Pre-op ROE Score**	**4.0 ± 3.0**	**4.0 ± 3.0**	**3.0 ± 7.0**	0.945
	3.0 [1.0; 5.0]	4.0 [2.0; 7.0]	4.0 [2.0; 4.0]	
**Post-op ROE Score**	16.0 ± 3.0	17.0 ± 2.0	16.0 ± 2.0	0.544
	16.0 [16.0; 17.0]	17.0 [15.0; 19.0]	15.0 [14.0; 18.0]	
***p*** **-value**	**<0.001**	**<0.001**	**<0.001**	

Values are presented as mean  ±  SD and median [interquartile range]; n: number. *p*-values were calculated with One-way ANOVA.

**Table 5:** Values of ROE change according to gender and BMI groups.

**Gender**	***Female (n = 12)***	**12.3 ± 3.2** **14.0 [9.0; 15.0]**
	***Male (n = 7)***	**12.3 ± 3.0** **12.0 [11.0; 14.0]**
***p*** **-value**		0.967 ^a^
**BMI**	*<18.5 (n = 1)*	14 ±. 14.0 [14.0; 14.0]
	*18.5–24.9 (n = 14)*	11.9 ± 3.2 11.5 [10.0; 14.0]
	*>25 (n = 4)*	13.3 ± 2.5 13.5 [11.5; 15.0]
***p*** **-value**		0.621 ^b^

Values are presented as mean  ±  SD and median [interquartile range]; n: number. *p*-values were calculated with ^a^ One-way ANOVA or ^b^ Kruskal–Wallis test.

## Data Availability

The datasets generated and/or analyzed during the present study are not publicly accessible because of the restricted license applicable to this research; however, they can be obtained from the corresponding author upon reasonable request.
